# Fat Inclusion Level, NaCl Content and LAB Starter Cultures in the Manufacturing of Italian-Type Ostrich Salami: Weight Loss and Nutritional Traits †

**DOI:** 10.3390/foods9040476

**Published:** 2020-04-10

**Authors:** Marco Cullere, Enrico Novelli, Antonella Dalle Zotte

**Affiliations:** 1 Department of Animal Medicine, Production and Health, University of Padova, Agripolis, Viale dell’Università, 16, 35020 Legnaro, Italy; marco.cullere@unipd.it; 2 Department of Comparative Biomedicine and Nutrition, University of Padova, Agripolis, Viale dell’Università, 16, 35020 Legnaro, Italy; enrico.novelli@unipd.it

**Keywords:** Italian-type salami, ostrich meat, sodium reduction, fat reduction, starter cultures, meat processing

## Abstract

The experiment studied the effect of two different fat inclusion levels (30% and 40%), NaCl contents (2.4 and 2.6%) and starter cultures (lactic acid bacteria (LAB) 6: *L. curvatus*/*S. xylosus*; LAB 8: *L. sakei*/*S. xylosus*) on the weight loss and nutritional composition of Italian-type ostrich salami. With this purpose, 8 batches of 9 salami each (*n* = 72) were prepared. Salami were ripened for 20 weeks: weight loss was monitored throughout the experiment, while salami nutritional composition was evaluated at 10 and 20 weeks of ripening. The lowest fat and highest salt inclusion levels provided the highest cumulative weight loss throughout the trial. At 10 weeks of ripening, salami with 40% fat were the richest in moisture and fat, whereas the leanest ones had the highest protein, ash and cholesterol contents. LAB 6 provided salami with the highest moisture and protein, while LAB 8 increased fat and cholesterol contents. At 20 weeks of ripening the proximate composition of ostrich salami was solely affected by fat inclusion level, with similar findings to those observed at 10 weeks. Overall, fat inclusion level had a great impact on the weight loss and nutritional composition of Italian-style ostrich salami. Reducing the NaCl inclusion from 2.6% to 2.4%, the weight loss of ostrich salami was retarded by approximately 1 week, without affecting the nutritional composition of the final product. Results of the study suggested that it is feasible to produce salami with lower fat and salt contents, while ensuring satisfactory product quality.

## 1. Introduction

Italian-type salami are intended to be slow ripened sausages, rarely smoked, with a pH not below 5 and generally between 5.3 and 6.2. They have been produced for centuries, starting from Roman times, and traditionally they are made out of pork meat and fat in variable ratios, salt, and eventually sugar and nitrate/nitrite [1]. As no starter cultures are used in the manufacturing of artisanal-made salami, the fermentation process is driven by autochthonous microflora and this originates a vast regional diversity which is typical of artisanal-made salami. Nowadays, starter cultures are being increasingly used in salami manufacturing as they ensure product safety and acceptable quality, together with reducing the ripening time [2]. Lactic acid bacteria (LAB) mainly ferment sugars into lactic acid, being thus responsible for the acidification of the product, but they generally lack the main aroma production pathways [3]. By contrast, coagulase negative cocci (CNC) such as *Staphylococcus* degrade free amino acids and inhibit the oxidation of unsaturated free fatty acids, ultimately contributing to the color and flavor formation in the salami. Among LAB species, *Lactobacillus* (*L*.) *sakei* is often the dominant one in traditional salami, followed by *L. curvatus* and *L*. *plantarum*. Considering CNC, *Staphylococcus xylosus* is reported to be the most common microbial species in Italian-type salami [4]. Overall, many studies have demonstrated that the aforementioned microbial species are suitable and adapted to the meat environment and ripening process with specificities linked to different ripening conditions, ingredients and meat species [5,6,7,8].

Salt (NaCl) is a key ingredient for salami manufacturing because it affects the final taste of the product, being a flavor enhancer, as well as the texture, together with ensuring microbiological stability mainly through water activity reduction [9]. For these reasons, in order to provide a satisfactory fermentation process and quality of the final product, its level should always be >2% [10]. Despite raw meat generally having a low NaCl content, meat products can provide 20–30% of NaCl dietary intake [11]. In salami manufacturing, fat can account for 40–50% of the final product and it has a pivotal role as it affects flavor formation, texture and color, thus guaranteeing satisfactory quality attributes.

In recent years, however, consumer and health organization concerns regarding products containing significant quantities of fat and salt have notably increased. The reduction of total fat intake, and more specifically the reduction of saturated fat intake, have been identified as a pivotal part in the prevention of unhealthy weight gain in adults and in the reduction of noncommunicable diseases [12]. In fact, obesity is associated with an increased risk of numerous cancers, coronary heart disease and strokes [13]. A reduction in salt consumption is also a key factor in lowering the risk of heart disease, strokes and hypertension [14]. The recommendations by the joint Food and Agriculture Organization of the United Nations (FAO)/World Health Organization (WHO) expert consultation on Fats and fatty acids in human nutrition point out the concerns regarding these pathological conditions, both in industrialized and middle-income countries. In an attempt to meet the nutrition and health needs, the new approaches to develop healthy food products, including fermented sausages, have been steadily growing [15,16,17].

On the basis of the aforementioned considerations, this research work aimed to study the effects of two different fat and salt inclusion levels and two different LAB starter cultures on the quality of an Italian-type slow-fermented salami manufactured with ostrich meat and evaluated at two different ripening times. Ostrich meat was chosen because it is a healthy alternative to pork meat thanks to its low fat, mainly polyunsaturated fatty acids, content [18]. Moreover, the meat of this species could replace pork meat in countries where ostriches are farmed on a large scale, thus possibly being used by the meat industry to develop new products that, potentially, could provide an added value to a local resource. This paper, which is the first part of a wide research project, focused on the weight loss and nutritional traits (proximate composition and cholesterol content) of the studied product.

## 2. Materials and Methods

### 2.1. Animal, Diet and Meat

The present work was a collaboration between the Department of Animal Medicine, Production and Health-MAPS of the University of Padova and the private farm “Azienda Struzzo 2000” (Volto, RO, Italy). For the experiment, a 90 kg male ostrich (*Struthio camelus*) was used. The ostrich was reared in a 100 m^2^ outdoor paddock of the aforementioned private farm where it was fed with a crumbled mix of 60% alfalfa (*Medicago sativa*), 20% of maize and 20% of fresh carrots provided by the horticultural production of the farm. The chemical composition of the ostrich diet is shown in Table 1. The ostrich was transported to the slaughterhouse where, according to the animal welfare dispositions [19], it was electrically stunned and bled. The carcass was then plucked, skinned, eviscerated and passed through a pre-cooling tunnel (+2 °C). Afterwards, it was cut in two halves (thighs) which were cooled for 24 h at +4 °C. The carcass was then dissected and, after bones, tendons and cartilage elimination, the meat was used for salami preparation.

### 2.2. Experimental Design and Salami Preparation

The present experiment was planned as a 2 × 2 × 2 design: 8 batches of 9 ostrich salami each (*n* = a total of 72 salami of approximately 650 g/each, fresh weight) using 2 different levels of fat (30% and 40%), NaCl (2.4% and 2.6%) and two different LAB starter cultures (LAB 6 and LAB 8) were produced. For the salami preparation, 30.5 kg of fresh ostrich meat and 16.5 kg of fresh pork back-fat were used. Meat and fat were ground separately by means of a professional meat grinder, equipped with a plate with 6 and 7 mm diameter holes, respectively. Subsequently, following the experimental design, ground meat and fat were divided into two batches according to two different fat inclusion percentages (30 and 40%) and then mixed. Subsequently, each batch was separated in two equal units and added with 2.4 or 2.6% of NaCl, followed by a spices mix (0.78% black pepper, 0.009% cinnamon, 0.009% cloves, 0.009% nutmeg) and 3.55% red wine. Each unit was finally split into two equal parts which were inoculated (1.6 g/kg) and mixed with two different LAB starter cultures: LAB 6 (*Lactobacillus curvatus*/*Staphylococcus xylosus* + dextrose) or LAB 8 (*Lactobacillus sakei*/*Staphylococcus xylosus* + dextrose). Batches were then individually mixed and put in a refrigerated chamber at +4 °C for 12 h. The following day all the batters were stuffed into natural casings (salami diameter ranged 6–8 cm), labeled and put in a dedicated chamber using controlled ripening conditions. All the salami were nitrite/nitrate free. During drying and ripening processes surface mold growth was not prevented, which is coherent with artisanal-type salami. Despite this, only a limited growth of white molds was observed along the process.

### 2.3. Drying and Ripening

Initially, salami were dried for a 5-day long period with RH (Relative Humidity) ranging from 65 to 85% and T (Temperature) starting from 19 °C and decreasing of 1 °C/day. When T was 14 °C, ripening phase started: RH was maintained between 70 and 80% and T decreased of 1 °C/day until 12 °C, afterwards it remained constant. Ripening of the first group of salami (4 salami/treatment) was stopped when the first of them lost up to 35% of its initial weight, at 10 weeks, while the second group of salami (5 salami/treatment) was ripened for further 10 weeks.

### 2.4. Weight Loss Determination and Chemical Analyses

Once a week, for 10 weeks, and once every two weeks, for the further 10 weeks, salami were individually weighed using a commercial weight scale and cumulative weight loss for each treatment was subsequently calculated. After collection, at 10 and 20 weeks of ripening, salami were transported to the MAPS Department, individually freed from casings, frozen in liquid nitrogen, homogenized using a Retsch Grindomix GM 200 (15 s at 10,000 rpm) and then analyzed. At 10 and 20 weeks of ripening, proximate composition was determined (dry matter: method 950.46; ash: method 923.03; crude fat: method 960.39), with protein content calculated by difference [20]. Cholesterol content of the salami was also analyzed at 10 and 20 weeks of ripening, following the method described by Casiraghi et al. [21].

### 2.5. Chemical Composition of the Diet

Analyses of the ostrich diet was carried out in duplicate following the Association of Official Analytical Chemists (AOAC) [22] methods to determine the concentrations of dry matter (method: 934.01), crude protein (method: 2001.11), crude fiber (method: 978.10), ash (method: 967.05) and starch (amyloglucosidase-α-amylase method: 996.11). Crude fat was determined after acid-hydrolysis [23]. Gross energy content of the diet was determined with an adiabatic bomb calorimeter [24]. Neutral detergent fiber (NDF, without sodium sulfite), acid detergent fiber (ADF), acid detergent lignin (ADL) and acid-insoluble ash (AIA) were analyzed according to Mertens [25], AOAC (procedure 973.187) [22] and Van Soest et al. [26], respectively, using the sequential procedure and the filter bag system (Ankom Technology, New York, NY, USA). Mineral analyses of the diet (Ca, P, Fe) was performed by ICP-OES (Spectro Ciros Vision EOP) after microwave digestion AOAC (procedure 999.10) [22].

### 2.6. Statistical Analysis

Data were analyzed using SAS 9.1 statistical analysis software for Windows [27] General Linear Model (GLM) procedures. A three-way ANOVA, which was stratified by ripening time (2.5 and 5 months), tested fat, salt and LAB starter cultures as fixed effects on cumulative weight loss, proximate composition and cholesterol content of artisanal-made Italian-type ostrich salami. The statistical analysis considered also interactions (Fat × Salt; Fat × LAB; Salt × LAB; Fat × Salt × LAB). When no significant interactions were found, only main effects were considered. Least square means were obtained using the Bonferroni test. *p*-values were considered significant when < 0.05.

## 3. Results

### 3.1. Weight Loss of Ostrich Salami

Weight loss of ostrich salami was significantly affected by fat (F) and salt (S) inclusion levels, from the beginning until the end of the ripening time (20 weeks), but not by LAB starter culture (L) (Table 2). After the drying period, salami manufactured with 30% fat already showed a more intense weight loss compared to those with 40% fat content (*p* < 0.0001).

**Figure 1:**
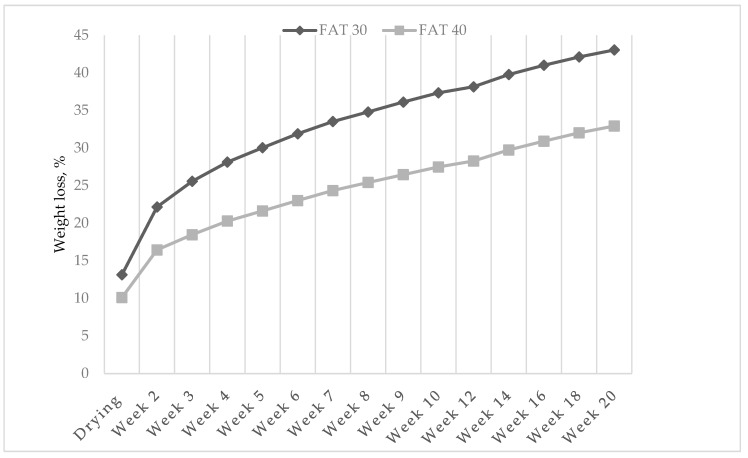
Effect of fat level on cumulative weight loss (% of the initial weight) of ostrich salami ripened for 20 weeks.

This difference tended to increase along the trial (Figure 1) and resulted in a 10% discrepancy in cumulative weight loss, in the two fat groups, at the end of the ripening phase (43.1 vs. 32.9% for F30 and F40 salami, respectively).

**Figure 2:**
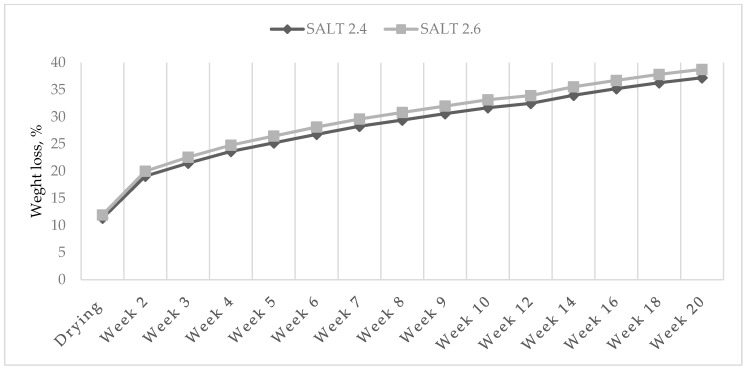
Effect of salt level on cumulative weight loss (% of the initial weight) of ostrich salami ripened for 20 weeks.

A higher NaCl content favored the weight loss of salami: in fact, it was higher in the 2.6% group compared to the 2.4% one (*p* < 0.05). In this case, the divergence between the two groups of salami increased from the drying phase until the fifth week of ripening, but after that it remained constant until the end of the trial (Figure 2).

**Figure 3:**
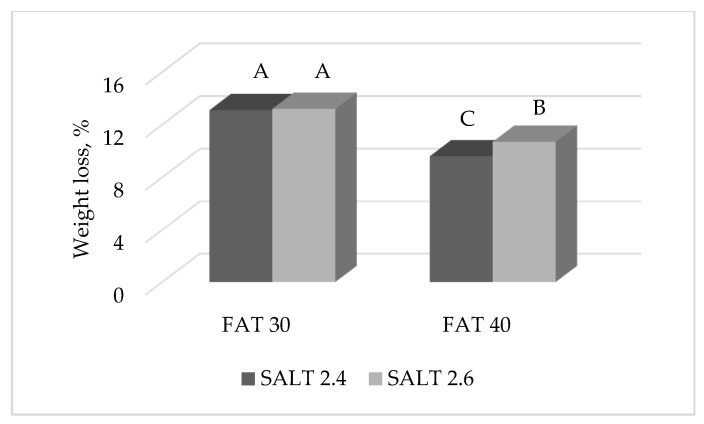
Effect of the interaction FAT × SALT on the cumulative weight loss (% of the initial weight) of ostrich salami at the end of the drying phase. Histograms reporting different A, B, C letters significantly differ for *p* < 0.05.

The F × S interaction showed an effect on the cumulative weight loss of ostrich salami: it was observed that within the group of salami with 30% fat, those manufactured with 2.4% and 2.6% salt inclusions exhibited the same cumulative weight loss, whereas at 40% fat inclusion level the salami with a higher salt content showed a more intense weight loss compared to those belonging to the 2.4% NaCl inclusion level (Figure 3).

**Figure 4:**
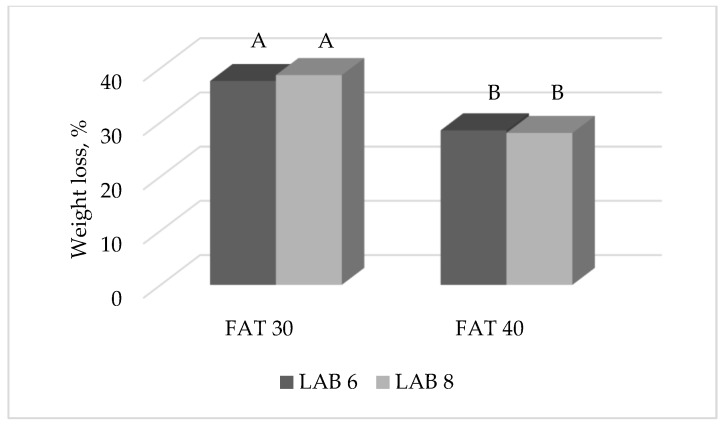
Effect of the interaction FAT × LAB on the cumulative weight loss (% of the initial weight) of ostrich salami ripened for 16 weeks. Histograms reporting different A, B letters significantly differ for *p* < 0.05.

Results of the present study highlighted also that different LAB starter cultures did not affect cumulative weight loss of ostrich salami over a 20 weeks ripening period. However, the F × L interaction highlighted a statistically significant difference for the cumulative weight loss up to 16 weeks of ripening (Figure 4; *p* < 0.05); despite this, no differences were observed between the two starter cultures within the FAT group.

### 3.2. Proximate Composition and Cholesterol Content of Ostrich Salami

The results presented in Table 3 show that fat level affected moisture, protein, fat, ash and cholesterol contents of Italian-style ostrich salami, analyzed at 10 weeks of ripening (*p* < 0.0001). As expected, higher presence of pork back-fat directly increased the fat content of the salami, which was higher in the 40% than the 30% groups (35.6 vs. 32.8 g/100 g, respectively). Interestingly, the leanest salami exhibited the highest cholesterol content (93.4 vs. 83.1 mg cholesterol/100 g meat, for 30 and 40% fat inclusion levels, respectively). Salami containing 30% pork back-fat had also lower moisture (33.2 vs. 36.4 g/100 g, respectively), higher protein (25.8 vs. 20.8 g/100 g, respectively) and ash (4.91 vs. 4.28 g/100 g, respectively) contents, compared to those manufactured with 40% back-fat.

Different salt percentages affected the ash content of ostrich salami, being the highest in the 2.6% salt group (4.86 vs. 4.34 g/100 g, respectively; *p* < 0.0001), whereas the other variables remained unaffected.

In the present trial, the use of different starter cultures had a considerable effect on the proximate composition of ostrich salami: when inoculated with LAB 8 salami showed lower fat (33.1 vs. 35.3 g/100 g, respectively) and cholesterol (85.7 vs. 90.8 mg/100 g, respectively) contents compared to those inoculated with LAB 6. By contrast, the presence of *L. curvatus* and *S. xylosus* lowered protein (23.1 vs. 23.5 g/100 g, respectively) and moisture (33.8 vs. 35.7 g/100 g, respectively) contents compared to salami inoculated with *L. sakei* and *S. xylosus*.

**Figure 5:**
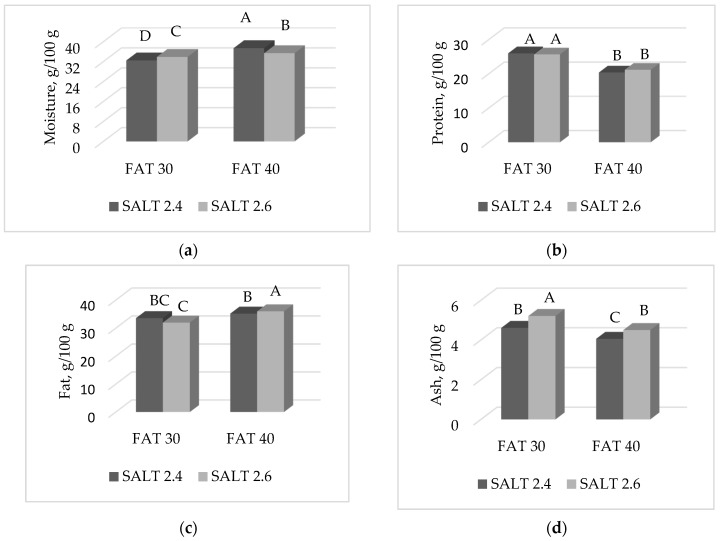
Effect of the interaction FAT × SALT on: (**a**) moisture (*p* < 0.0001); (**b**) protein (*p* < 0.05); (**c**) fat (*p* < 0.05); (**d**) ash (*p* < 0.05). Histograms reporting different A, B, C, D letters significantly differ.

A significant F × S interaction was observed for moisture (*p* < 0.0001), protein (*p* < 0.05), fat (*p* < 0.05) and ash (*p* < 0.05) contents of ostrich salami ripened for 10 weeks. When 2.4% salt was added, the moisture content of salami manufactured with 40% fat (Figure 5a) was higher compared to salami prepared with 2.6% salt (37.4 vs. 35.4%, for 2.4 and 2.6% Salt groups, respectively). However, when the fat content of salami was 30%, the situation was exactly the opposite with 2.4% Salt group showing a lower moisture content than the 2.6% Salt group (32.5 vs. 33.9%, for 2.4 and 2.6% Salt groups, respectively).

Protein content was the highest in 30% fat salami, with no differences between the two levels of Salt within the same Fat group (Figure 5b). Considering the 40% fat inclusion level, salami manufactured with 2.6% salt had a higher fat content compared to those having 2.4% salt; this difference, however, was not significant considering salami having 30% fat, as 2.4 and 2.6% salt levels showed similar fat values (Figure 5c). Ash content was higher in the low fat compared to high fat salami and, within each fat group, a higher presence of salt increased the ash content compared to salami manufactured with the lowest salt level (Figure 5d).

**Figure 6:**
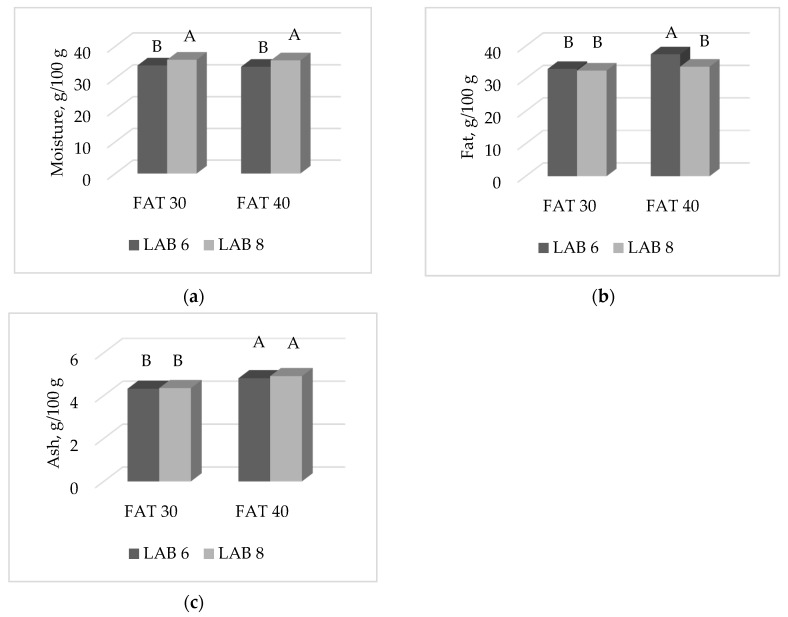
Effect of the interaction FAT × LAB on: (**a**) moisture (*p* < 0.0001); (**b**) fat (*p* < 0.01); (**c**) ash (*p* < 0.01). Histograms reporting different A, B letters significantly differ.

A significant F × L interaction was observed for moisture (*p* < 0.0001), fat (*p* < 0.01) and ash (*p* < 0.01) contents of ostrich salami ripened for 10 weeks (Figure 6a–c). Specifically, salami inoculated with LAB 6 starter culture showed a lower moisture content compared to those inoculated with LAB 8, both at 30 and 40% fat inclusion levels. Salami of the 40% Fat group inoculated with LAB 6 had the highest fat amount, with other combinations not differing among each other. Ash content was different and greater in high fat salami compared to low fat ones, with different starter cultures showing similar results within the same fat inclusion level (*p* < 0.01).

At 20 weeks of ripening, only fat content significantly affected the proximate composition of ostrich salami, whereas Salt and LAB starter cultures were ineffective (Table 4). Moisture content was higher in salami manufactured with 40% fat than those made with 30% fat (*p* < 0.0001); leaner salami had the highest protein (27.5 vs. 21.5 g/100 g for 30 and 40% fat inclusion levels, respectively), ash (5.54 vs. 4.79 g/100 g for 30 and 40% fat inclusion levels, respectively) and cholesterol (104.3 vs. 86 mg/100 g for 30 and 40% fat inclusion levels, respectively) contents. Conversely, the amount of fat was similar in salami manufactured either with 30 or 40% pork back-fat.

**Figure 7:**
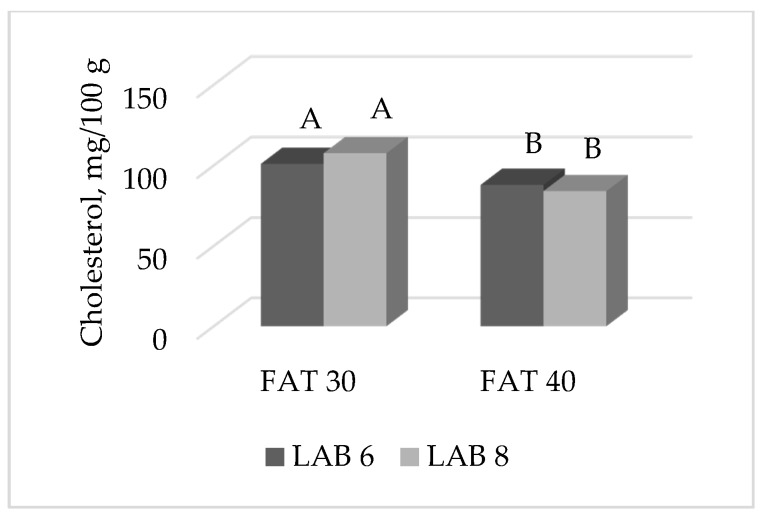
Effect of the interaction FAT × LAB on the cholesterol content (mg/100 g product) of ostrich salami ripened for 20 weeks. Histograms reporting different A, B letters significantly differ for *p* < 0.05.

The only interaction observed at 20 weeks of ripening was F × L on the cholesterol content (Figure 7): independently to the starter culture, salami manufactured with 30% fat had a higher cholesterol content than those with 40% fat. However, the histogram suggested a numerical difference between LAB 6 and LAB 8 within the same fat level: at 30% fat, LAB 6 had a lower cholesterol content than LAB 8 salami (101.0 vs. 107.6 mg/100 g for LAB 6 and LAB 8, respectively), whereas at 40% fat the opposite was observed (88.0 vs. 84.1 mg/100 g for LAB 6 and LAB 8, respectively).

## 4. Discussion

Lean meat contains more water than fat tissue, which explains why salami manufactured with a higher proportion of ostrich meat lost more weight compared to the fattest group of salami. Furthermore, fat has a crucial role for rheological and textural properties of meat products, especially when the product is manipulated thoroughly, as is the case of salami, as it binds water to form stable emulsions [28]. As a consequence, a lower fat content leads to a lower emulsion stability which causes a higher moisture loss [29]; the latter also contributes to explaining the findings of the present experiment. Similarly, Muguerza et al. [30] evidenced that weight losses of fermented sausages made of pork meat were significantly affected by fat level and that the lower the fat level the higher the weight loss of sausages. Moreover, at 28 days of ripening, the average weight loss of sausages manufactured with 30% pork back-fat was 38.5% which is much higher than the values observed in our study (28.1% of cumulative weight loss for salami belonging to the 30% fat inclusion level, at 4 weeks of ripening). As weight losses are known to depend on many processing factors such as temperature, relative humidity and air movement [31,32], this difference is not surprising as processing parameters differed in the two experiments.

The amount of water and its state in meat can change depending on numerous factors related to the tissue itself and to how the product is handled [33]. Salt (NaCl) is responsible for the solubilization of myofibrillar proteins in meat, which stimulates the proteins to increase hydration and water-binding capacity resulting in an improved texture, tenderness and juiciness [10]. Despite this, results of the present experiment highlighted that a higher NaCl content favored weight loss of ostrich salami. This finding could be explained by the fact that salt leads to the loss of free water too, which is also responsible for the decrease in water activity [34]. The different pattern in the observed salami weight loss as a result of the F × S interaction, indicated that, at 30% fat level, the 2.4% salt was enough to extract the free water from the salami, whereas at 40% fat only a higher salt concentration could reduce water availability as a higher stability of the emulsion fat-water was reached. Conversely, it is reasonable to expect that the ionic strength in the salami with 40% fat and 2.6% salt could reach values close to the threshold of the so called salting out (ionic strength >1 M), where water holding capacity starts to decrease thus favoring water loss [35].

The fact that different LAB starter cultures did not affect cumulative weight loss of ostrich salami was in line with the study by Kenneally et al. [36] on salami made of beef/pork meat inoculated with different microbial starters. Sometimes, in our study the interaction F × L on salami weight loss showed significant results (Table 2), but observing the histogram showing this interaction at 16 weeks of ripening (Figure 4) shows that different LAB starter cultures did not produce significant variations in terms of weight loss within the same fat inclusion level. For this reason, it was hypothesized that the significance of the interaction was mostly attributable to the different fat content rather than different LAB starter cultures. Trying to understand the reason for such interaction, authors have also considered the pH value of salami made with different starter cultures (Novelli et al., personal communication), a physical trait known to be strictly connected with water holding capacity. However, at 10 weeks of ripening salami belonging to LAB 6 and LAB 8 groups had an identical pH value (5.46), which confirmed the findings of this first part of the study: starter cultures were not a pivotal factor in affecting salami weight loss. Also, even if the pH value of the final product (20 weeks of ripening) slightly differed in salami manufactured with different starter cultures (5.47 for LAB 6 and 5.52 for LAB 8), this was not enough to determine diverse weight losses too.

Initial different fat contents changed the proportion of nutrients as well as the moisture content of ostrich salami, thus affecting the proximate composition of the product throughout the ripening process. Such findings could be explained by the fact that a lower fat content in ostrich salami determined a higher weight (water) loss during ripening, thus causing a higher concentration of nutrients compared to salami manufactured with 40% fat. This was the cause for the similar fat content observed at 20 weeks of ripening in the salami belonging to the two Fat groups. The clear different weight loss along the trial in salami belonging to 30% and 40% Fat groups, was also partially responsible for the highest cholesterol content observed in the 30% Fat salami. Moreover, as cholesterol is naturally present in the sarcolemma of muscle cells [37], a higher inclusion of lean meat from ostrich in our 30% Fat salami also explains the higher cholesterol content found in this group.

In the present study the salt content had a negligible effect on the proximate composition of ostrich salami and the different ash contents observed in the two groups was a direct consequence of the highest presence of salt. This finding was supported by results presented in a study evaluating the effect of NaCl partial substitution on proximate composition of Italian salami [11], where salami manufactured with 2.7% NaCl had the same proximate composition of those prepared with low NaCl formulations. Despite salt content of salami can affect the growth of starter cultures [38], results of the present experiment seemed to indicate that this was not a key factor neither for weight loss nor for the nutritional composition of ostrich salami as no interactions were observed between salt level and type of starter culture used.

Tissue lipase are primarily responsible for lipolysis during the fermentation process, but it has now been accepted that staphylococci can act as lipolytic bacteria, thus playing an important role in aroma formation [39]. Similarly, in the first stages of ripening, proteolysis in meat is due to endogenous enzymes such as calpains and cathepsins which break sarcoplasmic and myofibrillar proteins, while it is in the last stages of ripening that microbial enzymes play a predominant role in the secondary hydrolysis of oligopeptides and small peptides [40]. However, an appropriate choice of a combination of strains in the formulation of a starter culture is fundamental for successful fermentation and ripening processes, as different strains and microbial species are known to act differently according to different meat types, technological characteristics of the fermentation and ripening parameters [8,38,41,42]. In the current research, the presence of *L. curvatus* and *S. xylosus* (LAB 6) led to salami characterized by lower protein and moisture contents compared to those inoculated with *L. sakei* and *S. xylosus* (LAB 8). This could be the consequence of a different intensity in the degradation of the meat proteins, possibly as a combination of endogenous and microbial enzymes; in fact, the water activity of the two groups of salami, also analyzed at 10 weeks of ripening, differed, as well as their content of nonprotein nitrogen (Novelli et al., personal communication). This hypothesis found confirmation in the results of a study on the proteolytic activity of LAB [43], showing that different enzyme combinations from *L. sakei* and *L. curvatus* provided different results: the first exhibited exopeptidase activity and the second modifying the peptide profile. In a study considering the effect of the starter culture on proteolytic changes during processing of fermented sausages [44], it was observed that sausages inoculated with *L. sakei* or *L. carnosus* differed in terms of some individual amino acids. However, different from the processing stage, which was the main factor affecting proteolytic process, the type of starter culture was not a factor affecting total free amino acids concentration. In the present study, as a result of their metabolic activity, different LAB starter cultures generated salami with different nutritional composition until 10 weeks of ripening, whereas at 20 weeks the concentration of salt was probably too high for their survival [45], thus their activity might have been reduced or completely hampered. In fact, it was demonstrated that growth and survival of staphylococci, but especially of LAB, is markedly affected by NaCl concentration [6].

From results of the present study it emerged that ostrich salami manufactured with 30% pork back-fat and inoculated with different starter cultures showed the same fat content, whereas at 40% fat inclusion level, the combination *Lactobacillus curvatus*/*Staphylococcus xylosus* provided salami with a higher fat content compared to those containing *Lactobacillus sakei*/*Staphylococcus xylosus*. This finding suggested that the significance of the main effect of the starter culture on the fat content of ostrich salami was particularly evident when the fat level of the salami was 40%, whereas at 30% inclusion percentage only a tendency was observed. As no specific studies evaluating the effect of different fat contents on the growth of starter cultures for the production of fermented sausages have been conducted until now, further research on this topic is required; this is because the choice of the appropriate starter mix for each specific fermented meat product is of fundamental importance to ensure a satisfactory product quality [46].

## 5. Conclusions

Fat inclusion level had a great impact on the weight loss and nutritional composition of Italian-style ostrich salami, independent of the ripening phase. A lower fat content consistently shortened ripening time, thus being a positive aspect in terms of productivity, and it determined a higher nutrients concentration compared to high fat salami, with the only drawback of a higher cholesterol content compared to high fat salami. Reducing the NaCl inclusion from 2.6 to 2.4% retarded the weight loss of ostrich salami by approximately 1 week, without affecting the proximate composition and cholesterol content of the final product. At 10 weeks of ripening, *L. sakei* provided salami with a healthier nutritional composition compared to salami inoculated with *L. curvatus*. The metabolic activity of tested LAB starter cultures seemed to be affected by the fat inclusion level, even if further investigations to clarify this point is necessary. Understanding the latter could help to ensure a high quality product.

## Figures and Tables

**Table 1:** Chemical composition (g/kg as is basis) of the ostrich diet.

Analyzed Composition	g/kg
Water	126
Crude protein	147
Crude fat	17.8
Ash	60.7
Crude fiber	143
Neutral detergent fiber	231
Acid detergent fiber	155
Acid detergent lignin	22.7
Acid Insoluble Ash	4.74
Starch	213
Gross Energy, MJ/kg	8.90
Ca	10.5
P	2.50
Fe	0.12

**Table 2:** Effect of two levels of fat, salt, and lactic acid bacteria (LAB) starter cultures and their interaction on cumulative weight loss (% of the initial weight) of ostrich salami ripened for 20 weeks ^1^.

	FAT (F)		SALT (S)		LAB (L)		Significance					RSD ^2^
	30	40	2.4	2.6	6	8	F	S	L	F × S	F × L	
Drying	13.2	10.1	11.3	11.9	11.5	11.7	<0.0001	<0.01	ns	<0.05	ns	0.65
Week 2	22.2	16.5	19.1	20.0	19.4	19.7	<0.0001	<0.001	ns	<0.05	ns	0.86
Week 3	25.6	18.5	21.5	22.6	21.9	22.2	<0.0001	<0.001	ns	<0.01	ns	0.92
Week 4	28.1	20.3	23.6	24.8	24.1	24.3	<0.0001	<0.001	ns	<0.05	<0.05	0.98
Week 5	30.1	21.6	25.2	26.5	25.7	26.0	<0.0001	<0.0001	ns	<0.05	<0.05	0.98
Week 6	31.9	23.0	26.8	28.1	27.3	27.6	<0.0001	<0.05	ns	ns	ns	1.95
Week 7	33.5	24.4	28.3	29.6	28.9	29.0	<0.0001	<0.0001	ns	<0.05	<0.05	1.04
Week 8	34.8	25.4	29.4	30.8	30.0	30.2	<0.0001	<0.0001	ns	<0.05	<0.05	1.04
Week 9	36.1	26.5	30.6	32.0	31.2	31.4	<0.0001	<0.0001	ns	<0.05	<0.05	1.06
Week 10	37.4	27.5	31.7	33.2	32.3	32.5	<0.0001	<0.0001	ns	<0.05	ns	1.06
Week 12	38.2	28.3	32.5	33.9	33.1	33.4	<0.0001	<0.0001	ns	<0.01	<0.05	0.95
Week 14	39.8	29.8	34.0	35.6	34.6	35.0	<0.0001	<0.0001	ns	<0.05	<0.05	0.97
Week 16	41.0	30.9	35.2	36.7	35.8	36.2	<0.0001	<0.0001	ns	<0.05	<0.05	0.97
Week 18	42.1	32.0	36.3	37.8	36.8	37.3	<0.0001	<0.0001	ns	<0.01	ns	0.99
Week 20	43.1	32.9	37.2	38.8	37.8	38.2	<0.0001	<0.0001	ns	<0.05	ns	1.01

ns = not significant; ^1^ Up to 10 weeks of ripening the sample size was of 9 salami/treatment, while from 10 to 20 weeks of ripening it was 5 salami/treatment; ^2^ Residual Standard Deviation.

**Table 3:** Effect of two levels of fat, salt, and two LAB starters cultures and their interaction on proximate composition (g/100 g) and cholesterol content (mg/100 g) of ostrich salami ripened for 10 weeks.

	FAT (F)		SALT (S)		LAB (L)		Significance					RSD ^1^
	30	40	2.4	2.6	6	8	F	S	L	F × S	F × L	
Salami	4	4	4	4	4	4						
Moisture	33.2	36.4	34.9	34.6	33.8	35.7	<0.0001	ns	<0.0001	<0.0001	<0.0001	0.36
Protein	25.8	20.8	23.2	23.4	23.1	23.5	<0.0001	ns	<0.05	<0.05	ns	0.55
Fat	32.8	35.6	34.4	34.0	35.3	33.1	<0.0001	ns	<0.001	<0.05	<0.01	1.37
Ash	4.91	4.28	4.34	4.86	4.57	4.63	<0.0001	<0.0001	ns	<0.05	<0.01	0.07
Cholesterol ^2^	93.4	83.1	88.6	87.9	90.8	85.7	<0.0001	ns	<0.001	ns	ns	3.83

ns = not significant; ^1^ Residual Standard Deviation; ^2^ Cholesterol content of the batter was 58.2 mg/100 g.

**Table 4:** Effect of two levels of fat, salt, and two LAB starters cultures and their interaction on proximate composition (g/100 g) and cholesterol content (mg/100 g) of ostrich salami ripened for 20 weeks.

	FAT (F)		SALT (S)		LAB (L)		Significance				RSD ^1^
	30	40	2.4	2.6	6	8	F	S	L	F × L	
Salami	5	5	5	5	5	5					
Moisture	26.4	31.1	29.0	28.5	28.0	29.5	<0.0001	ns	ns	ns	2.61
Protein	27.5	21.5	24.3	24.7	24.2	24.8	<0.0001	ns	ns	ns	1.18
Fat	36.5	36.5	36.4	36.6	37.1	35.9	ns	ns	ns	ns	3.58
Ash	5.55	4.79	5.07	5.28	5.22	5.13	<0.0001	ns	ns	ns	0.42
Cholesterol	104.3	86.0	95.7	94.7	94.5	95.9	<0.0001	ns	ns	<0.05	6.54

ns = not significant; ^1^ Residual Standard Deviation.
